# Optimized Roflumilast Ethosomal Gel for Enhanced Transdermal Therapy in Atopic Dermatitis: An In Vitro Study

**DOI:** 10.1002/open.70335

**Published:** 2026-11-11

**Authors:** Yasir Mehmood, Syeda Momena Rizvi, Atika Javed, Rida Siddique, Hamza Rafeeq, Hira Shahid, Ousman Brahim Mahamat, Hafiz Muhammad Zohaib, Khalid S. Almaary, Gamal A. Shazly, Musa A. Said, Turki M. Dawoud

**Affiliations:** ^1^ Riphah Institute of Pharmaceutical Sciences (RIPS) Riphah International University Faisalabad Pakistan; ^2^ Department of Biochemistry Faculty of Sciences University of Agriculture Faisalabad Faisalabad Punjab Pakistan; ^3^ Department of Biochemistry Riphah International University Faisalabad Pakistan; ^4^ Department of Pharmacology Faculty of Pharmaceutical Sciences GC University Faisalabad Faisalabad Pakistan; ^5^ National Federation of Associations of Medical Practitioners in Chad (FENAPMT) Ministry of Health N’Djamena Republic of Chad; ^6^ Institute of Biopharmaceutical and Health Engineering Tsinghua Shenzhen campus Tsinghua University Shenzhen China; ^7^ Department of Botany and Microbiology College of Science King Saud University Riyadh Saudi Arabia; ^8^ Department of Pharmaceutics College of Pharmacy King Saud University Riyadh Saudi Arabia; ^9^ Department of Chemistry Faculty of Science Islamic University of Madinah Madinah Saudi Arabia

**Keywords:** atopic dermatitis, design expert, ethosomes, permeability, roflumilast, transdermal

## Abstract

This study evaluated a transdermal drug delivery system using roflumilast (RT)‐loaded ethosomal gel for the topical treatment of inflammatory skin disorders. RT‐loaded ethosomes were prepared using the cold method and incorporated into a topical gel. Eight formulations were developed and optimized using a Design of Experiments approach to investigate the effects of formulation variables on drug entrapment and performance. Among them, RT‐5 was selected as the optimized formulation and evaluated for physicochemical properties, stability, microbiological quality, drug release, and transdermal permeation. RT‐5 exhibited suitable topical characteristics, including a pH of 5.3–5.5, viscosity of 90–91 cP, and good spreadability. In vitro diffusion studies at pH 5.5 showed 25.5% ± 3% drug release within the first hour compared with 5.2% ± 2% from the commercial cream. Moreover, RT‐5 achieved cumulative drug penetration of 99.34% after 12 h, demonstrating enhanced transdermal delivery. Stability studies showed that the formulation maintained acceptable pH, viscosity, consistency, and appearance for 6 months under recommended storage conditions and at least 3 months under accelerated conditions. Microbiological testing revealed no detectable bacterial growth. The outcome of this work showed that RT‐5 demonstrated satisfactory stability, quality, and improved transdermal delivery of roflumilast.

## Graphical abstract

Roflumilast‐loaded ethosomal gel was developed and optimized using a Design of Experiments approach for enhanced transdermal delivery. The optimized RT‐5 formulation exhibited suitable pH, viscosity, spreadability, stability, and microbiological quality, achieving 99.34% drug penetration after 12 h, demonstrating promising topical delivery potential.

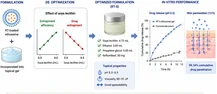

## 1 Introduction

Atopic dermatitis (AD) is a common form of eczema that causes itchy, dry, and cracked skin. AD is the most prevalent form of eczema in children and frequently appears before their first birthday. However, it can also emerge in adults for the first time. AD treatments usually include topical application of an ointment or lotion. According to current recommendations, mild or transient AD should be treated with class II creams, while moderate or recurrent AD should be treated with class III creams [1]. Some of the creams couldn’t penetrate the skin due to the large molecular size, and incomplete systemic absorption of the active pharmacological component may lead to decreased pharmacokinetic effectiveness when applied topically [2, 3].

RT inhibits phosphodiesterase‐4 (PDE4) specifically and over a longer period of time. There was a time when it was used systemically to treat atopic dermatitis (AD). However, that was only on a localized basis [4, 5]. For the treatment of plaque psoriasis, a 0.15% roflumilast cream has been developed, authorized by the FDA, and is now on the market. It has a good safety record and proven clinical effectiveness [6]. Roflumilast (RT) is a selective phosphodiesterase‐4 (PDE4) inhibitor. Its chemical name is 3‐(cyclopropylmethoxy)‐N‐(3,5‐dichloropyridin‐4‐yl)‐4‐(difluoromethoxy)benzamide. It has molecular formula C_17_H_14_Cl_2_F_2_N2O_3_ and molecular weight of about 403.2 g/mol. Roflumilast is a compound consisting of a substituted benzamide attached to a dichloropyridine ring and cyclopropylmethoxy and difluoromethoxy groups [7]. RT has its own constraints. A significant limitation is that the chemical DEs do not dissolve in water, necessitating the use of potentially hazardous organic solvents and oils for solubilization. Moreover, when in contact with organic solvents, RT becomes unstable, which makes RT a more complex molecule to form a cream or ointment [8]. To ensure that RT is cost‐effective, improvements in its water solubility and stability are needed. The topical application of medication has been a longstanding practice to achieve various effects at different levels of the skin. Several issues have been identified with traditional topical treatments, including limited absorption through the skin barrier and the potential for systemic absorption. Several new methods for skin delivery of active pharmaceutical ingredients are currently described in the scientific literature [9]. Innovative systems composed of natural lipid materials, such as solid lipid nanoparticles (SLNs) and nanostructured lipid carriers (NLCs), are among these choices. NLCs and SLNs have attracted the attention of several research groups for potential topical applications [3]. Because of their small size, they can be applied directly to the skin, improving drug absorption. Improved skin hydration is a result of lipid nanoparticles’ occlusive characteristics. Furthermore, chemicals that are susceptible to photolysis, oxidation, and hydrolysis can have their chemical stability enhanced by lipid nanoparticles [10]. In recent years, the emergence of biomimetic materials has shifted the paradigm in skincare science [11]. A lot of work has been done in the previous 20 years on these techniques [12]. They are suitable for topical, dermal, and transdermal applications. The transdermal route is widely used to deliver chemical drugs and natural compounds to treat skin conditions (e.g., skin diseases, premature aging) because of its numerous advantages over the oral (per os) route, including avoidance of first‐pass effects, decreased fluctuation in drug plasma level, (local) targeted delivery leading to lower side effects and increased efficiency, and increased patient compliance [13, 14]. Microneedle‐assisted nanomedicine delivery is also considering good candidate for skin penetration, but there are some limitations of this dosage form. However, there are some limitations of ethosomes also. Ethosomes are lipid‐based deformable vesicular carriers mainly composed of phospholipids, ethanol, and an aqueous phase. The high ethanol content enhances the membrane fluidity and deformability, thus enabling the vesicles to interact with and penetrate the stratum corneum more effectively. These properties can enhance drug solubilization, skin deposition, and transdermal permeation. Thus, ethosomes are a promising carrier for drugs with poor aqueous solubility like roflumilast.

We have used specialized formulations called ethosomes to encapsulate the active ingredient (RT) within a lipid layer, thereby enhancing transdermal drug delivery by preventing premature dissolution and increasing penetration [15, 16]. RT‐based ethosomes were developed to achieve high skin penetration with the aim of increasing the drug’s bioavailability for the treatment of atopic dermatitis [17]. The Strat‐M platform was used to test the efficacy of RT in traversing a synthetic artificial membrane. Strat‐M is a synthetic membrane intended to mimic the texture and appearance of real skin. During penetration testing on both people and animals, it might be used as a substitute for actual skin [18]. To improve the penetration of transdermal drug delivery, the present work primarily focused on developing and studying a new polymeric ethosomes gel for RT delivery. First and foremost, this study aimed to construct a model to investigate which variables regulate ethosomes’ gel entrapment efficiency. Methods related to ethosomes gel formation and drug entrapment were examined using a DE strategy to optimize formulations by systematically determining the impacts and interactions of these factors. We used the concentration of soya lecithin and the ethanol content as our two formulation parameters. We used carbopol polymer in this ethosomes gel formulation; it is a conductive polymer, and these polymers are softer and exhibit swelling properties [19, 20].

## 2 Materials and Methods

### 2.1 Chemicals

Dae‐Jung Chemicals & Metals in Siheung‐si, South Korea, supplied our lab with soy lecithin (soy lecithin was used as the phospholipid source for ethosome preparation, containing approximately 25% phosphatidylcholine (PC), propylene glycol, 99.99% ethanol, Carbopol 934, and triethanolamine. Saffron Pharmaceutical of Faisalabad, Pakistan, supplied the 99% pure roflumilast used in the experiments. This investigation used distilled water. Merck Millipore, Burlington, USA, supplied the synthetic membrane (Strat‐M) used to measure roflumilast absorption through the skin.

### 2.2 Roflumilast Loaded Ethosome Preparation

According to Supraja R. and Sailaja A.K [21, 22, 23]. The organic and water‐based phases were manufactured separately. The organic phases of soya lecithin (SL) and RT (50 mg) were dissolved in ethanol at room temperature at 1000 rpm with a magnetic stirrer. Propylene glycol (PG) was dissolved in distilled water to create the aqueous phase. Then, using a syringe, the aqueous phase was gradually introduced to the organic phase while being continuously stirred to promote the creation of ethosomal vesicles. To create a homogeneous ethosomal suspension, the resultant mixture was agitated for 30 min at 1000 rpm.

A probe sonicator (VCX 750, Sonics and Materials Inc., USA) operating at 20 kHz was used to sonicate the suspension for 5 min while maintaining a temperature of 4 °C to prevent thermal damage. The ethosomes suspension obtained after sonication was kept at 4 °C for further examination. Before adding the ethosomes to the gel formulation, a number of characterization tests were carried out to ensure their stability and preparedness.

### 2.3 Preparation of Roflumilast‐Loaded Ethosomal Gel

Carbopol 934 was used as a gelling agent to create the ethosomal gel. To achieve full hydration and the creation of a homogeneous gel basis, 1 g of Carbopol 934 was dissolved in 100 mL of distilled water and continuously swirled for 2 h. One gram of the previously made RT‐loaded ethosomal solution was added to the gel base while being continuously mixed after Carbopol 934 had fully hydrated. Then, as a formulation component, 5 mL of propylene glycol (PG) was added. 0.5 mL of triethanolamine (TEA) was added to the mixture to bring its pH down to about 5.5. To create a homogeneous ethosomal gel, the final formulation was homogenized using a high‐shear homogenizer (Ultra‐Turrax T25, IKA, USA) at 12,000 rpm for 5 min. Carbopol 934 was tested at concentrations of 1%, 2%, and 3% w/v to maximize the gel’s consistency and rheological properties. To find the ideal Carbopol content for the final RT‐loaded ethosomal gel formulation, the produced formulations were next assessed for viscosity and other pertinent physicochemical properties.

## 3 Experimental Design

A statistical design technique based on response surface methodology (RSM) was used to formulate and optimize RT‐loaded ethosomal gels. The impact of two crucial formulation variables, the concentration of ethanol and soy lecithin, was examined using a face‐centered cubic central composite design. To ensure repeatability and statistical reliability, these two independent variables were varied across eight experimental runs, with each formulation being prepared in triplicate. The drug’s entrapment efficiency (%) was chosen as the primary dependent variable to be optimized. To separate the effects of lecithin and ethanol, the amounts of propylene glycol (5 mL) and RT medication (50 mg) were maintained constant throughout all formulations. To anticipate the optimal formulation conditions, a second‐order polynomial equation was fitted to the experimental data using stepwise least‐squares regression. Fivefold cross‐validation, one‐way analysis of variance (ANOVA), and lack‐of‐fit tests at a 95% confidence interval were used to evaluate the model’s robustness. These statistical instruments demonstrated the model’s importance, precision, and predictive capacity. The interplay between lecithin and ethanol concentrations and their impact on entrapment efficiency was also shown using three‐dimensional response surface plots, which provided important new insights into the formulation behavior of the ethosomes system (Table 1).

**TABLE 1:** Composition of the formulation of RT‐loaded ethosomes.

Ethosomes formulation	Lecithin (Soya lecithin, mL)	Ethanol, mL	Propylene glycol, mL	**Drug, mg** **RT**
RT‐1	0.50	1.00	5	50
RT‐2	4.00	1.00	5	50
RT‐3	0.50	5.00	5	50
RT‐4	4.00	5.00	5	50
RT‐5	4.72	3.00	5	50
RT‐6	2.25	0.17	5	50
RT‐7	2.25	5.83	5	50
RT‐8	2.25	3.00	5	50

### 3.1 Evaluation of Ethosomes Loading Capacity and Drug Entrapment Effectiveness

A Waters Breeze HPLC system (Waters, Milford, USA) comprising a Waters 1525 binary pump with an in‐line degasser, a Waters 717 Plus autosampler, and a Waters 2487 dual‐wavelength UV absorbance detector was used to perform the high‐performance liquid chromatography (HPLC) study. At room temperature, chromatographic separation was accomplished using a Kromasil C18 column (250 mm × 4.0 mm i.d., 5 µm particle size; Tedia, Brazil) under isocratic conditions [24, 25]. Methanol and sodium tetrabutylammonium hydrogen sulfate were combined in a 60:40 (v/v) ratio to form the mobile phase. Phosphoric acid was used to lower the mobile phase pH to 5.0–5.5. At a wavelength of 248 nm, detection was carried out while the flow rate was kept constant at 1.0 mL/min [26, 27]. Equations (1) and (2) were used to determine the entrapment efficacy and drug loading of ethosomes containing roflumilast [28, 29].

(1) $$\text{Entrapment efficiency}\;\%\frac{W_{t}‐W^{f}}{W_{t}}\times 100$$

where:

*W*_t_ = Total amount of roflumilast in the formulation

*W* ^f^ = Amount of unencapsulated/free roflumilast

(2) $$\text{Drug loading \% =}\frac{\text{Mass of drug}\;\text{in Ethosome}}{\text{Mass of Ethosome recovered}}\text{× 100}$$

### 3.2 Analysis of Ethosomes Using Atomic Force Microscopy and Scanning Electron Microscopy

The surface morphology of the ethosomes solution loaded with roflumilast was examined using SEM. This included measures of roundness and smoothness and the development of ethosome aggregates. We selected the formulation that was most effective in trapping the medicines for SEM imaging. The RT‐5 formulation was the most optimized for SEM.

### 3.3 Evaluation of Dispersity, Size, and Potential of Particles

The Malvern Nano‐ZetaSizer, developed by Malvern Instruments of Worcestershire, UK, was used to assess the size and zeta potential of the ethosome vesicles. Because it influences skin–vesicle interactions and vesicular qualities like stability, this parameter is critical. The zeta‐sizer and the possibility of drug entrapment were factors in our selection of the best formulation. In terms of optimizing zeta‐strength, zeta‐potential, and polydispersity index measurements, RT‐5 proved to be the most successful formulation [30, 31].

### 3.4 Analysis of Ethosomes Using Fourier Transform Infrared Spectroscopy

One tactic is to learn the relationships between the pure component groups and the structural groups that are of interest. The functional groups and interactions of roflumilast, soya lecithin, and RT‐5 ethosomes formulations were investigated. Using attenuated total reflection technology and a Bruker FTIR (Tensor 27 Series‐Bruker Corporation, Germany) instrument, a spectrum scan was accomplished within the region of 4000 to 800 cm^−1^ [21, 32, 33].

### 3.5 Analysis of Ethosomes Using Differential Scanning Calorimetry

A DSC Q200 TA from TA Instruments (Elstree, United Kingdom) was used to perform the measurements in triplicate. In 40 µL aluminum pans with three vent holes, samples ranging in weight from 2.5 to 3.5 mg were inserted. Zinc (419.7 C) and indium (156.6 C) were used as melting‐point standards to calibrate the device for temperature [34]. Using a modulation amplitude of ±0.796 and a modulation time of 60 s, materials were studied over a temperature range of 0–350 °C at a heating rate of 20 °C/min. Melt‐quenching was used to determine the glass‐transition temperature of the Roflumilast raw material. The sample was heated to 170 °C and then cooled down quickly. Version 4.7A of the Universal Analysis program was used to analyze the data [16, 21, 34, 36].

### 3.6 X‐Ray Powder Diffraction (XRPD)

The X‐ray powder diffraction patterns were acquired using a Rigaku Miniflex II desktop X‐ray diffractometer from Tokyo, Japan, and a Haskris WA1 cooling unit from Elk Grove Village, Illinois, USA. The patterns were obtained on a two‐theta (*θ*) scale with a step size of 0.05° per second, spanning from 3° to 40°. Three samples underwent analysis [37].

### 3.7 Release Study of Ethosomes

Using a USP Dissolution Apparatus II, the drug’s dissolution was investigated at pH of 5.5, which is the pH of the skin. The ethosomes suspension was placed in dialysis membrane pouches and combined with 900 mL of the dissolution liquid (buffer, pH 5.5), and the mixture was continuously spun at 50 rpm to maintain a constant drug concentration in the dissolution medium. Additionally, a pure Roflumilast water suspension was subjected to the same procedure, with the dissolving medium temperature set to 37 °C. As planned, we collected samples at 1, 2, 3, 4, 6, 8, 10, and 12 h, continuously substituting the measured volume with new material, and HPLC was used to measure the roflumilast release at 24 h [38]. The following formula was used to determine the percentage release (3)

(3) $$\begin{array}{l} \mathrm{Cumulative}\;\mathrm{drug}\;\mathrm{Release}\;(\%)\\ \;\;=\frac{V_{0}C_{n}+V_{s}(C_{1}+C_{2}\ldots \ldots .C_{n‐1})}{M}\times 100 \end{array}$$

where:

*C* *_n_* = drug concentration in the release medium at the current sampling time

*C*_*i*_ = drug concentration measured at each previous sampling time

*V*_0_ = total volume of the release medium

*V*_s_ = volume of sample withdrawn at each sampling time

*M* = total amount of drug initially present in the formulation.

### 3.8 Hemolytic Investigations of Ethosomes

We isolated RBCs by centrifuging a 5 mL sample of human blood at 3000 rpm for 5 min. Using a 4% phosphate buffer, the RBCs were carefully rinsed. Analyzed samples were incubated in blood tubes containing ethylenediaminetetraacetic acid (EDTA) at 37 ± 1 °C for 4 or 12 h after the ethosomes solution was mixed at different concentrations. Afterward, the mixture was spun in a tube containing EDTA at 1500 rpm for 5 min. The residual liquid’s absorbance was measured at 541 nm after the supernatant was removed. A positive control was Triton X‐100, while a negative control was phosphate‐buffered saline. Equation (4) was used to evaluate hemolysis in red blood cells. With the approval of the Ethical Review Board of Rashid Latif College of Pharmacy (IRB No. RLCP/EP/12/2024; the studies were conducted in accordance with the World Medical Association’s Declaration of Helsinki. The participants were apprised of the study’s goals and the intended use of their samples prior to their express agreement being given in Equation (4).

(4) $$\text{\% Hemolysis}=\frac{\text{ABS sample }-\text{ ABS blank}}{\text{ABS positive }-\text{ ABS blank}}\times 100\%$$

### 3.9 In Vitro Permeability Study of Ethosomes

A transdermal Franz diffusion sampling device was utilized to examine ethosomes’ skin penetration ex vivo. The device has an effective permeation area of 4.25 cm^2^ and a receptor cell capacity of 25 mL. At 37 °C, the phosphate‐buffered saline (receiver vehicle) with a pH of 5.5 was stirred at 50 rpm using a magnetic stirrer. Penetrance studies were performed to ascertain the efficacy of ethosomes loaded with RT in traversing the synthetic artificial membrane Strat‐M. Strat‐M is an artificial membrane that mimics the texture and appearance of real skin. The donor compartment was attached to a receptor compartment, and the stratum corneum side of the artificial skin was positioned toward it. The donor compartment was supplemented with one gram of weighed ethosomes. The drug content was assessed by taking several samples from the receptor compartment via the sampling port over 12 h. After each sample collection, the receptor phase was supplemented with a fresh volume of buffer equal to the original volume (Xingyan et al., 2011).

### 3.10 Rheological Study of Ethosomes Gel

An important rheological variable is viscosity, which is the fluid’s resistance to flow and is measured in centipoise. We measured the room‐temperature viscosity of the ethosomes gels at various rotating speeds using Spindle No. 7 and a Brookfield viscometer.

### 3.11 The Spreading Ability and pH of the Ethosomes Gel

Two slides were placed between them and subjected to a specific load; the time it takes for them to slide off the gel is a measure of a substance’s spreadability. The upper plate had a 20 g weight attached, and the glass slide was 6 cm long. The improved spreadability is directly proportional to the rate at which the two slides are separated. The following equation was used to determine the spreadability according to Equation (5)

(5) S=M/T

where *S* stands for the spreadability in g/s, *M* for the mass in g, and *T* for the duration in seconds.

One gram of ethosomes gel was dissolved in 30 milliliters of distilled water (pH 7.0–7.5) to determine the pH. By simply inserting the probe of a digital pH meter into the ethosomes gel, we were able to determine its pH.

### 3.12 Physical Stability and Microbial Assessment of Ethosomes

The ethosomes gel was tested for accelerated stability over a 6‐month period at 40 ± 2 °C and 75 ± 5% relative humidity. The first, third, and sixth months were the times of physicochemical examination, which included evaluations of microbiology, smell, pH, and viscosity.

### 3.13 Analytical Statistics

Graph‐Pad Prism v.5 ran several statistical tests, including Tukey’s test and ANOVA. We represented the data (SD) using the mean and standard deviation. The threshold for statistical significance was set at *p* = 0.05 [39].

## 4 Results and Discussion

### 4.1 Metabolic Engineering and Assessment of RT‐Loaded Ethosomes Reaction Time (RT) Entrapment and Loading Efficiency as a Function of Soya Lecithin

A range of soya lecithin concentrations was used in the cold procedure to prepare RT‐loaded ethosomes (Tables 2 and 3). When producing ethosomes on a large scale, this is one of the easiest approaches to use in the lab [40]. Soya lecithin was used as the principal lipid component in this experiment because of the medium’s low hydrolysis rate. During ethosomes preparation, the propylene glycol ratio remained fixed, while the RT/soya lecithin ratio was changed between 1 and 8 milliliters. When the ratio of soya lecithin to ethanol was adjusted, the drug entrapment efficiency ranged from 19.12 ± 0.4 to 70.22 ± 0.4, as shown in Table 3. By using optimal amounts of soy lecithin and ethanol, the entrapment efficacy was increased. The remarkable encapsulation efficiency seen during formulation may be attributed to the hydrophobic character of RT, which makes it nearly insoluble in water but soluble in lipids. This property likely allows for its simple absorption into the lipid bilayers of ethosomes (Figure 1) [41].

**TABLE 2:** Formulation variables and their levels.

Factor	Symbol	Unit	Low (−1)	Medium (0)	High (+1)
Soya lecithin	X_1_	mL	0.50	2.25	4.00
Ethanol	X_2_	mL	1.00	3.00	5.00
Roflumilast concentration	X_3_	mg	5.00	10.00	15.00

**TABLE 3:** Drug entrapment and drug loading (%) of formulations.

Formulation	Soya lecithin X_1_, mL	Ethanol X_2_, mL	Drug concentration X_3_, mg	Y_1_ Entrapment efficiency, %	Y_2_ Drug loading, %
RT‐1	0.50	1.00	10.00	19.12 ± 0.40	23.22 ± 0.50
RT‐2	4.00	1.00	10.00	57.36 ± 0.60	26.16 ± 0.60
RT‐3	0.50	5.00	10.00	20.21 ± 0.20	12.24 ± 0.60
RT‐4	4.00	5.00	10.00	67.26 ± 0.70	34.16 ± 0.20
**RT‐5**	**4.00**	**3.00**	**15.00**	**70.22 ± 0.40**	**39.32 ± 0.20**
RT‐6	4.00	3.00	5.00	52.22 ± 0.40	27.32 ± 0.20
RT‐7	0.50	3.00	15.00	47.22 ± 0.40	35.32 ± 0.20
RT‐8	4.00	3.00	15.00	48.22 ± 0.40	36.32 ± 0.20
RT‐9	2.25	1.00	5.00	31.45 ± 0.50	21.15 ± 0.40∗
RT‐10	2.25	5.00	5.00	42.68 ± 0.60	24.32 ± 0.30
RT‐11	2.25	1.00	15.00	48.56 ± 0.50	33.45 ± 0.40
RT‐12	2.25	5.00	15.00	55.74 ± 0.60	36.28 ± 0.50
RT‐13	2.25	3.00	10.00	68.22 ± 0.50	38.15 ± 0.30
RT‐14	2.25	3.00	10.00	69.35 ± 0.40	38.72 ± 0.30
RT‐15	2.25	3.00	10.00	70.22 ± 0.40	39.32 ± 0.20

*Note*: * indicates a statistically significant difference at P < 0.05.

**FIGURE 1:**
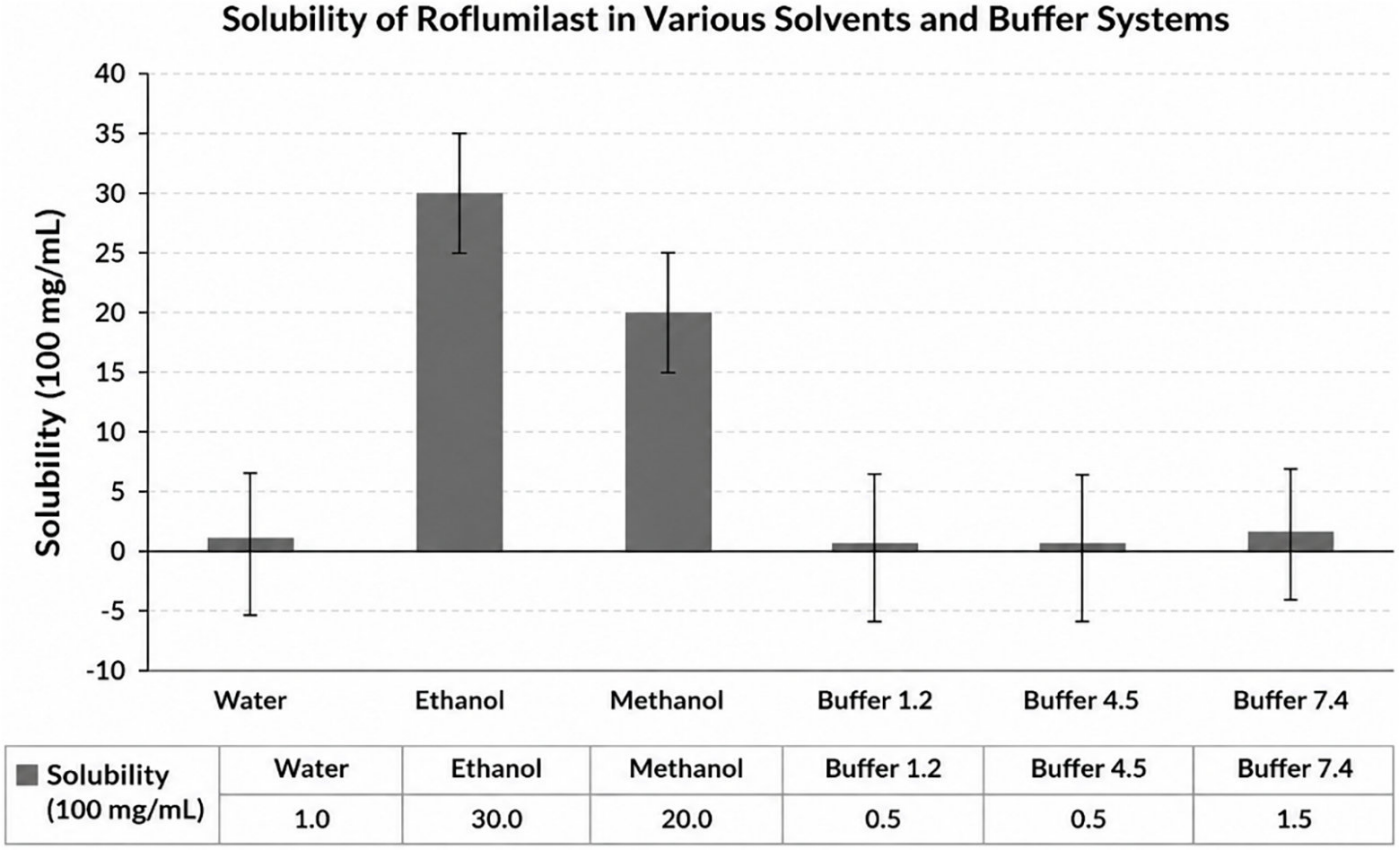
Solubility of RT in different solvents.

### 4.2 Roflumilast Solubility Study

The solubility of RT in selected organic solvents and aqueous buffer media was determined to investigate the solvent‐dependent solubility characteristics of RT and to help in the selection of a suitable vehicle for formulation development. A known weight of RT was added to each selected solvent or buffer and mixed well under controlled conditions to equilibrium. Samples were processed and filtered as appropriate, and the concentration of the dissolved drug was measured by the analytical method described in the study. Solubility was expressed as the amount of RT dissolved in unit volume of the respective solvent or medium. The solvents and media examined were ethanol, methanol, distilled water, pH 1.2 buffer, pH 4.5 buffer, and pH 7.4 buffer. The solubility values obtained were used for comparative evaluation and are presented in Figure 1. Because RT‐restricted aqueous solubility may limit its dissolution and administration in traditional aqueous formulations, these findings are crucial for formulation development. The creation of lipid‐based or vesicular delivery systems, such as ethosomes, can take advantage of the increased solubility of organic solvents. Appropriate solvent systems may accelerate drug incorporation and boost subsequent skin distribution. The RT‐5 ethosomal formulation showed increased solubilization of RT when compared to pure medication. The improvement may be related to the synergistic action of ethanol, phospholipids, and the nanoscale vesicular structure of the formulation. However, as an ethanol‐only control with the same ethanol content was not assessed, the contribution of ethanol per se to the observed solubility enhancement cannot be differentiated quantitatively from that of the whole ethosomal system. Therefore, the observed enhancement is due to the whole formulation rather than just the ethosome creation.

### 4.3 Statistical Modeling for Optimizing the RT‐Loaded Ethosomes Gel

The Design of Experiments approach was adopted to investigate the influence of formulation variables on the performance of RT‐loaded ethosomes. Three quantitative formulation variables were considered: soya lecithin concentration (X_1_), ethanol (X_2_), and roflumilast (X_3_). The responses of interest were entrapment efficiency (Y_1_) and drug loading (Y_2_). The formulation variables were studied at preselected levels, and the experimental responses were analyzed by Design‐Expert software (Version 12.0, Stat‐Ease Inc., Minneapolis, MN, USA). Analysis of variance (ANOVA) was employed to find the statistical significance of the fitted model and contribution of individual formulation variables. Response‐surface methodology was applied for evaluating the main, interaction, and quadratic effects of the formulation variables on the selected responses.

### 4.4 Effect of the Formulation Parameters on the Entrapment Efficiency

The entrapment efficiency of RT‐loaded ethosomal formulations was found in the range of 19.12 ± 0.40%–70.22 ± 0.40%, showing large variation in the drug incorporation among the formulations studied. This variation indicates that the composition of the ethosomal system had a major impact on the ability of the vesicles to incorporate roflumilast. The lowest entrapment efficiency was observed for RT‐1 (19.12 ± 0.40%), whereas the highest entrapment efficiency was achieved for RT‐5 (70.22 ± 0.40%). The approximately 3.7‐fold difference between these formulations indicates a significant improvement in drug incorporation under the formulation conditions, as represented by RT‐5. Soya lecithin is an important structural component of ethosomes; it provides the phospholipid matrix required for the formation of the vesicles. Thus, variations in the concentration of phospholipids can affect the availability of the lipid phase for incorporation of the lipophilic drug. Roflumilast is poorly soluble in water and moderately lipophilic; a suitable phospholipid environment may facilitate its insertion into the vesicular membrane. Ethanol is also an important factor in the formation of ethosomes. Ethanol, at the right concentration, can increase the fluidity and deformability of phospholipid membranes, possibly facilitating drug incorporation and vesicle–skin interaction. However, the effect of ethanol is concentration‐dependent, and excessive ethanol may increase the permeability of the membrane and possibly facilitate drug leakage. In this case, the effect of ethanol is more meaningful in combination with phospholipid and the drug concentration than the isolated effect of ethanol. To visualize the combined effects of the formulation variables, a response‐surface analysis was used. The contour and three‐dimensional response‐surface plots show the variations in the predicted entrapment efficiency over the studied formulation space. These plots are a graphical representation of the region of the formulation with a higher or lower incorporation of the drug (Figure 2).

**FIGURE 2:**
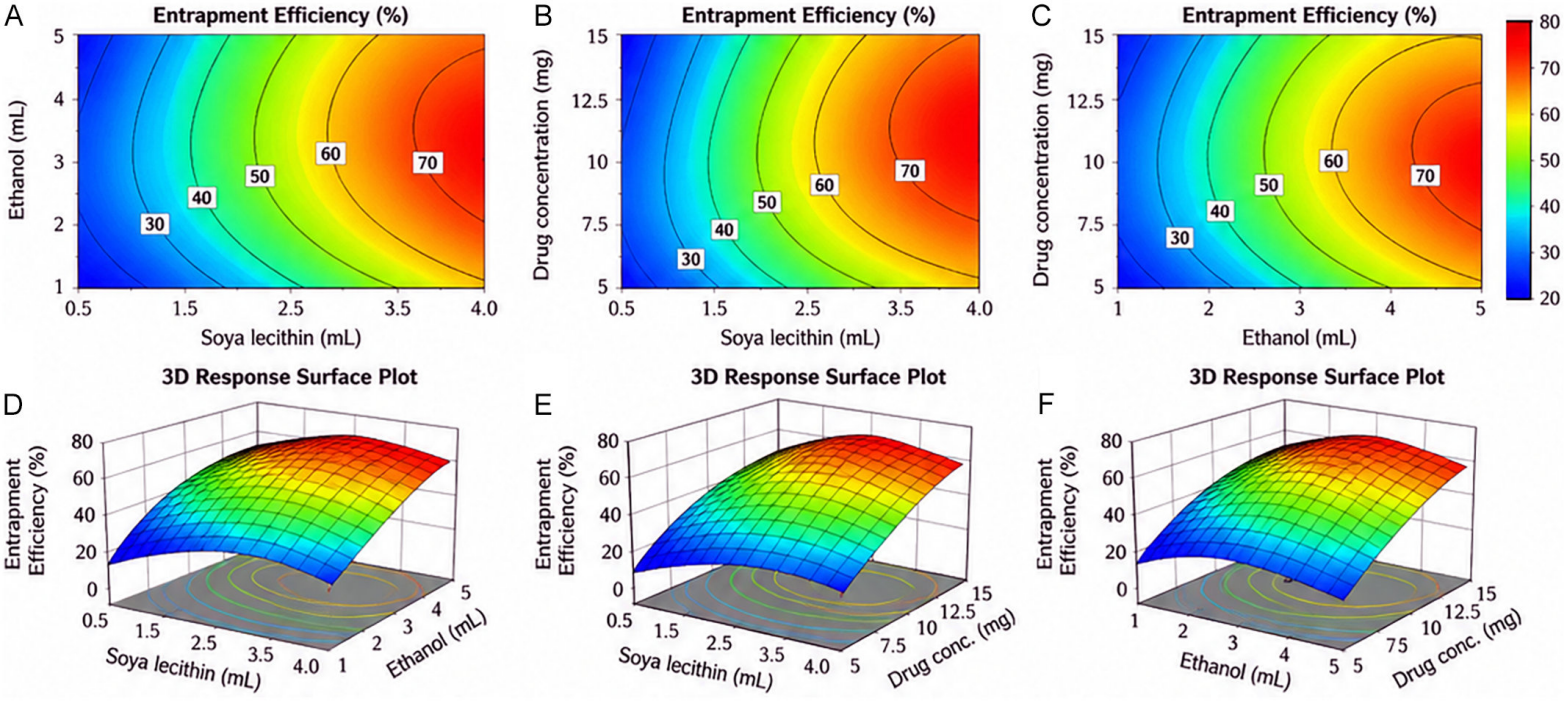
Contour plots showing the effects of the independent variables on encapsulation efficiency (Y1).

(6) $$\mathrm{DE}=43.91+20.32X_{1}+0.49X_{2}$$

### 4.5 Effect on Drug Loading

Also, significant differences were observed in the drug loading of the formulations, which ranged from 12.24 ± 0.60 to 39.32 ± 0.20%. RT‐3 showed the lowest drug loading, while RT‐5 showed the highest drug loading of 39.32 ± 0.20%. An important basis for the selection of RT‐5 for further studies was the simultaneous observation of the highest entrapment efficiency (70.22 ± 0.40%) and highest drug loading (39.32 ± 0.20%) in RT‐5. This combination suggests that RT‐5 was able to incorporate a relatively larger proportion of the drug and, at the same time, associate a higher amount of drug with the vesicular system. Note that the entrapment efficiency and drug loading are different properties of the formulation. Entrapment efficiency is the percentage of the starting drug that ends up in the vesicular formulation, and drug loading is the amount of drug in the formulation relative to the formulation components. Both responses were thus taken into account for the choice of the formulation to be further characterized.

#### 4.5.1 Evaluation of Medication Entrapment Effectiveness

After the ethosomes were broken, the best formulation was extracted, weighed, and dissolved in a 30:70 ratio of ethanol to newly made pH 7.4 phosphate buffer (USP) to create a suspension of RT ethosomes. Fifteen minutes of sonication at room temperature were applied to the mixture. We used a mobile phase to dilute the generated solution and then ran it by HPLC at 248 nm to determine the RT concentration [42]. The same methodology was used to examine each formulation, RT‐1 through RT‐8. Table 3 shows the results of loading and trapping of drugs. Using 4.7 mL of soya lecithin and 3 mL of ethanol, the RT‐5 formulation demonstrated substantial drug entrapment. Additional classifications will make use of this formulation.

#### 4.5.2 Scanning Electron Microscopy

Scanning electron microscopy (SEM) was used to examine the particle shape and diameter after the powder was manufactured [43]. Figure 3 displays four representative photos, labeled A, B, C, and D. Because of their spherical form and average diameter ranging from 0.87 ± 0.2 to 1.23 ± 0.3 μm, the ethosome particles we discovered are morphologically and dimensionally ideal for the most effective skin deposition (Figure 3B).

**FIGURE 3:**
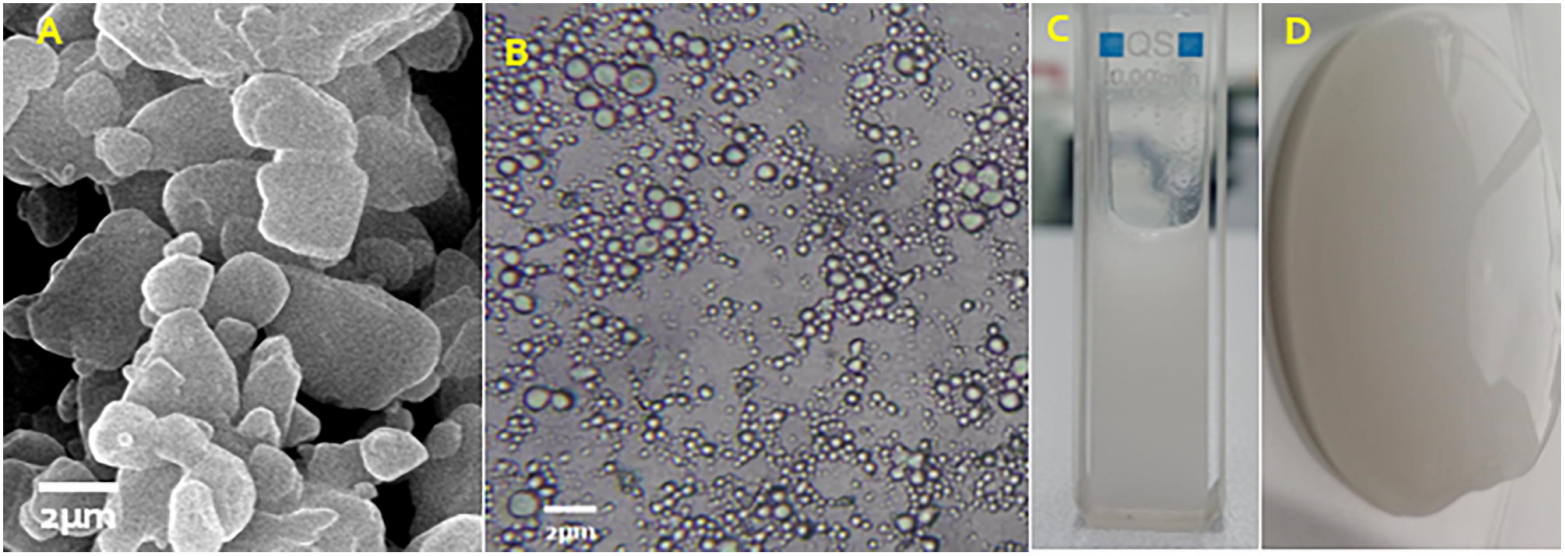
SEM images of the raw materials (A) and SEM image of the RT‐5 ethosomes (B). Camera picture of RT‐5 ethosomes suspension (C) and the gel (D).

The surface and morphological details of the pure RT and RT‐5 ethosomes were obtained by SEM, as shown in Figure 3. Pure RT exhibited a crystalline structure, as shown in Figure 3A, with a typical prism surface and an average particle size of 5 μm. It appears that the vesicle’s surface has been uniformly smoothed, making it more spherical. One possible explanation is that the vesicles containing the carbopol and RT complex disappeared because of the topological changes observed in the RT‐5 ethosomes. These results were consistent with the diffractograms generated from the XRD data.

### 4.6 Determination of Polydispersity Index and Particle Size

The research was conducted using an avalanche photodiode detector and a Malvern Nano‐ZetaSizer (Nano‐ZS, Malvern Instruments, UK) equipped with a 633 nm He–Ne laser. While the optimized RT ethosomes formulation was diluted in ethanol, measurements were done in triplicate at room temperature. The ethosomes RT‐5 have a high entrapment effectiveness (70.22 ± 0.4) and an average particle size (750nm ± 23), as shown in Figure 4. Particle size significantly affects how well drugs are absorbed through the skin. With a charge of −11 ± 4, the resultant EP had a PDI of 0.22 ± 0.32. Because of their influence on in vivo performance, stability, efficacy, and safety, particle size and polydispersity index (PDI) are crucial aspects for characterizing particles [44]. A very uniform formulation is indicated by an ethosomes PDI (particle size dispersion index) below 0.5 [45]. When the PDI is near 0.2, ethosomes are assumed to be homogeneous and composed of phospholipid vesicles [46].

**FIGURE 4:**
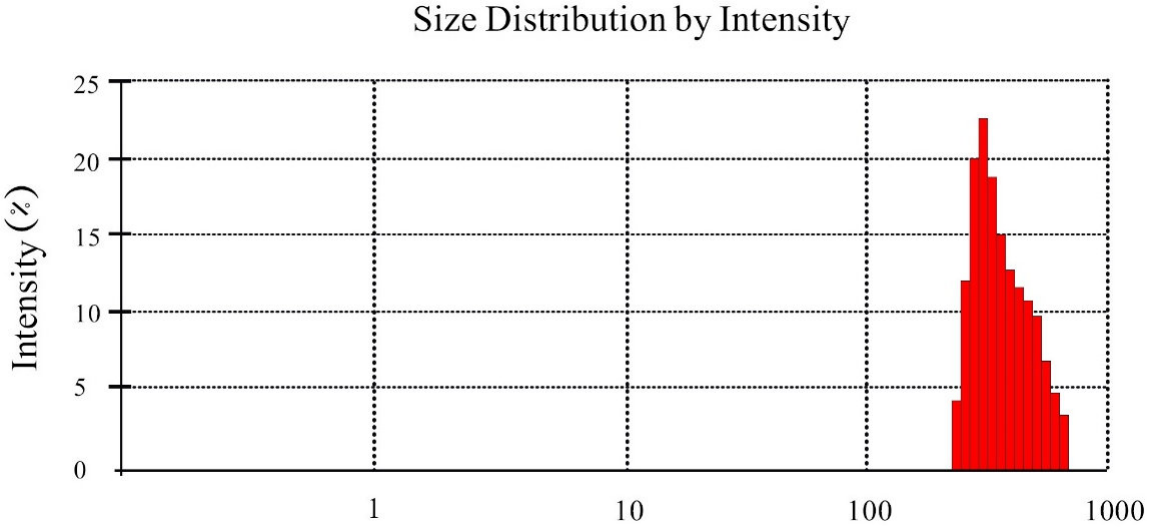
Size distribution of the RT‐5 ethosomes suspension.

#### 4.6.1 Fourier Transform Infrared Spectroscopy

The FTIR spectra of soy lecithin, RT, propylene glycol, and RT‐5 ethosomes are displayed in Figure 5. To examine each sample, we scanned it over the 800–4000 cm^−1^ range. The primary RT peaks for these wavenumbers are shown in Figure 5: N─H stretching, 3254 cm‐1; C─H stretching in the aromatic group; cyclopropy, 3093.7 cm^−1^; symmetrical and asymmetrical stretching vibrations of the CH2 aliphatic group; 2929.7 cm^−1^, stretching vibrations of C═O in the amide group; 1654 cm^−1^, stretching vibrations of C═C in the aromatic compound; 1401.5 cm^−1^, symmetrical stretching of N─C═O; 1558 cm^−1^, deformation vibration of the CH2 group; 1274.5 cm^−1^, symmetrical stretching of C─O─C in the cyclopropyl group; 1006.4 cm^−1^, asymmetric stretching of CF2; 1206.2 cm^−1^, out‐of‐plane vibration of CH in the aromatic compound; and 872–805 cm^−1^. Soya lecithin’s FTIR spectra show the following significant bands: (i) The vibrations at 2854, 2928, 2956, and 1462 cm^−1^ represent the symmetric CH2, antisymmetric CH2, and CH2 scissoring vibrational modes, respectively; (ii) The vibration at 1736 cm^−1^ represents the carbonyl stretching; and (iii) The infrared active vibrations of the highly overlapped PO2 − and P─O─C are located in the region between 1200 and 970 cm^−1^, with a center at around 1061 cm^−1^.

**FIGURE 5:**
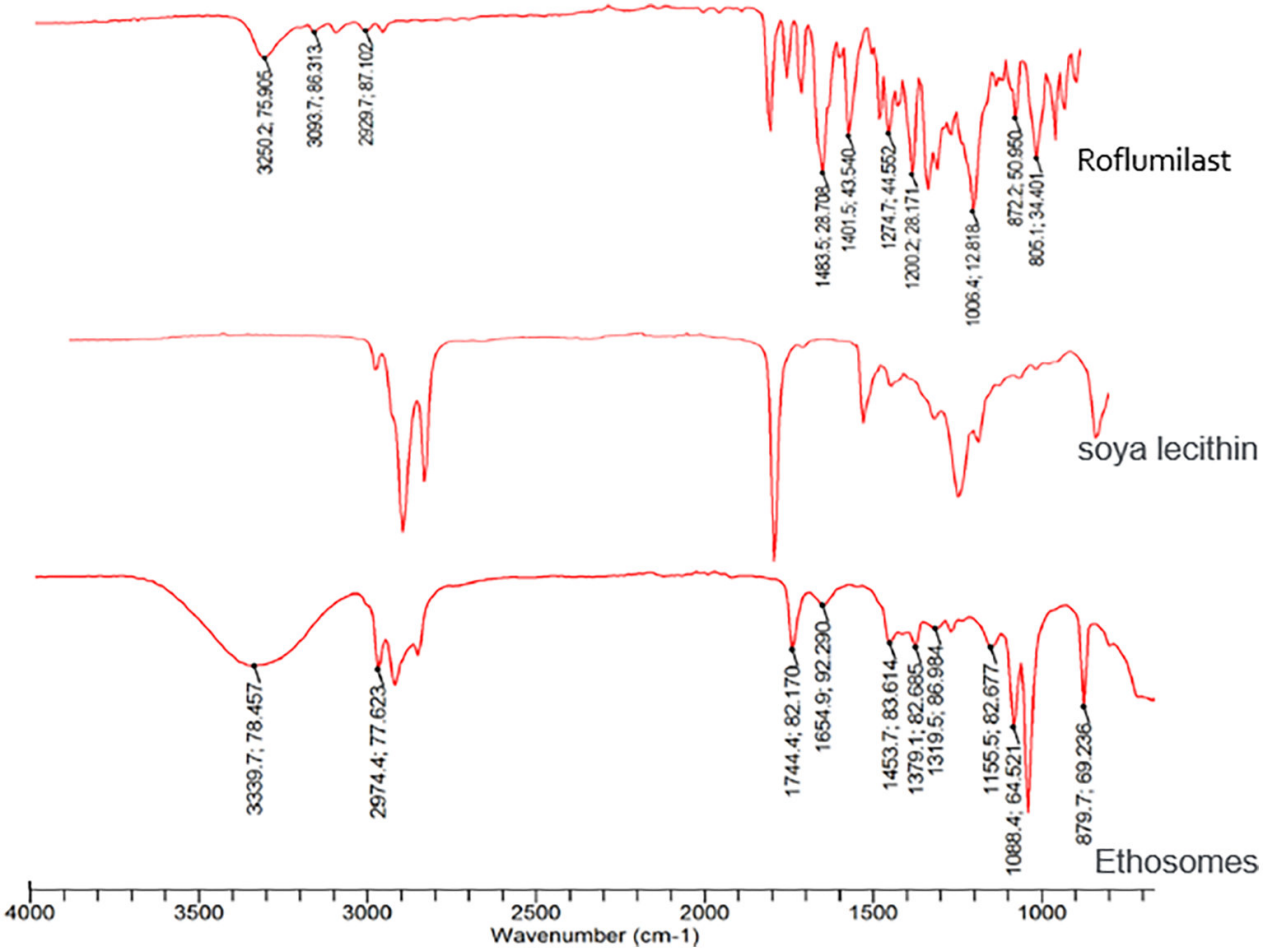
FTIR spectra of RT, lipids (soya lecithin), and ethosomes.

See Figure 5 for the FT‐IR spectra of the drug‐prepared ethosomes and RT. There were no chemical interactions observed in the FT‐IR spectra of RT with ethosomes as the binary mixtures exhibited all the expected peaks for both RT and ethosomes, with no noticeable peak shifts.

### 4.7 DSC Analysis

The thermal behavior of roflumilast (RT), soya lecithin, and RT‐5 ethosomes was examined using a TA Instruments Q2000 differential scanning calorimeter. Under the experimental conditions outlined in the Materials and Methods, the samples were examined over a temperature range of 0–400 °C at a heating rate of 20 °C/min. Figure 6 displays the relevant DSC thermograms, and Table 4 summarizes the enthalpy of fusion (ΔH_f_), peak temperature (T_peak_), and onset temperature (T_onset_). In line with the thermal behavior previously documented for RT, the DSC thermogram of pure RT showed two discrete endothermic transitions at roughly 59.55 °C and 171.8 °C (Ali, Faraat, et al.) [28].

**FIGURE 6:**
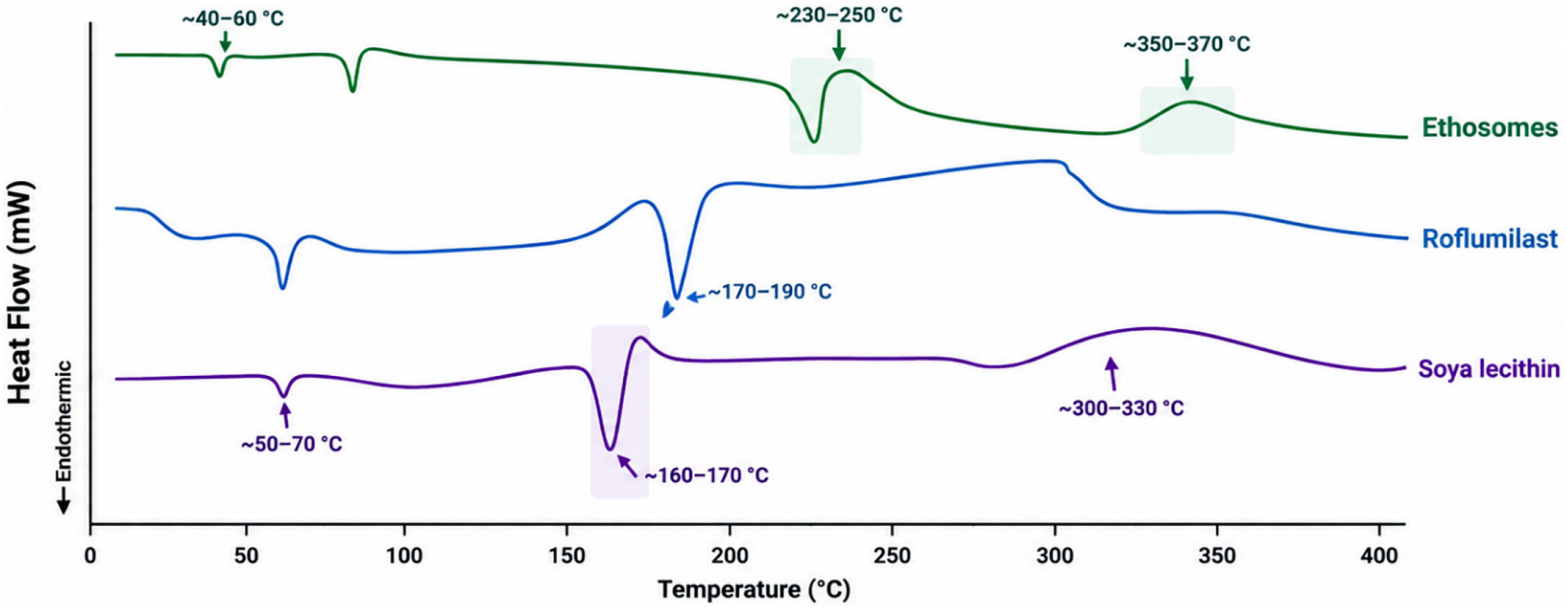
DSCs of RT, lipids (soya lecithin), and ethosomes.

**TABLE 4:** Peak temperature and enthalpy values of RT, ethosomes, and soya lecithin (*n* = 3).

Sample name	T_onset_/C	T_peak_/C	DH_f_/Jg^−1^
RT	59.55 °C	171.8 °C	3.51 ± 2
Soya lecithin	100.2 °C	160.9 °C	4.12 ± 2
Ethosomes	50.88 °C	228.12 °C	2.12 ± 3

At 160.9 °C, soy lecithin showed a noticeable endothermic transition that matched its distinctive thermal transition. In contrast, the RT‐5 ethosomal formulation did not exhibit the characteristic transition of soy lecithin as a clear, abrupt peak at the same temperature. This alteration could be linked to the phospholipid’s molecular organization and changed temperature environment during the creation of the hydrated, ethanol‐containing vesicular system. As a result, after inclusion into the ethosomal structure, the distinctive phospholipid transition may widen, change, or become less noticeable.

Thermal transitions were observed in the RT‐5 ethosomal formulation at roughly 50.88 °C, 75.23 °C, and 228.12 °C. While the major transition at 228.12 °C happened at a temperature significantly higher than the typical RT transition at 171.8 °C, the transitions around 50.88 °C and 75.23 °C may be related to the thermal behavior of the volatile and formulation compo‍nents, specifically ethanol and propylene glycol. The change in the RT‐associated thermal transition indicates that the ethosomal system’s molecular architecture and thermal environment were modified by the addition of RT. Nevertheless, this observation is interpreted as a change in thermal behavior after formulation because the DSC measurements do not prove the establishment of a particular chemical interaction.

In the ethosomal formulation, a broad thermal event was also noted at higher temperatures. This broadening could be a result of the several formulation components’ overlapping thermal transitions or decomposition‐related processes. Therefore, rather than being exclusively attributed to bond cleavage within the drug, polymer, or lipid network, this region should be viewed cautiously.

### 4.8 X‐Ray Diffraction (XRD)

X‐ray scattering measurements were conducted to analyze the solid structure of RT, soya lecithin, and the resulting formulations. Figure 6 displays the X‐ray diffractograms of the formulations under investigation (RT‐5) and their individual components. Figure 7 clearly shows that RT has multiple sharp peaks, suggesting its crystalline nature. The XRD pattern of the sample showed distinct peaks at 6.00°, 17.05°, 23.15°, and 24.15°, with corresponding counts of 308, 120, 175, and 493, respectively. Conversely, the diffractogram of soya lecithin indicated its amorphous nature. The RT peaks in the diffractogram of the pure drug became sharper (Figure 7). However, when mixed with polymers and lipids, a loss of sharpness was observed, possibly indicating a decrease in crystallinity and revealing the amorphous nature of ethosomes.

**FIGURE 7:**
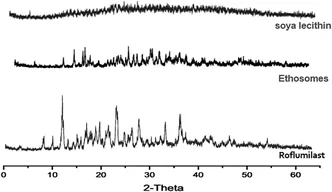
DSCs of RT, lipids (soya lecithin), and ethosomes.

### 4.9 Drug Release Study

Over the course of 12 h at pH 5.5, the in vitro release patterns of roflumilast (RT) from the optimized ethosomal gel, commercial RT cream (White petrolatum, cetostearyl alcohol, isopropyl palmitate, cetearyl phosphate, methylparaben, propylparaben, sodium hydroxide, and purified water), and pure RT were assessed. Based on its formulation properties, the optimized ethosomal formulation was chosen for the release investigation. Samples were taken at predefined intervals, and a Shimadzu HPLC system with UV detection at 248 nm was used to measure the amount of released RT. The release profiles of the three formulations varied significantly, as seen in Figure 8. When compared to both the commercially available cream and pure RT, the RT‐5 ethosomal gel showed a significantly greater and longer release profile. Over the course of the 12 h trial period, the ethosomal formulation demonstrated an initial release phase, followed by a steady and continuous rise in drug release. According to the plotted profile, about 20% of RT was released in the first hour, followed by progressive release that reached about 94% at 10 h and almost full release by 12 h.

**FIGURE 8:**
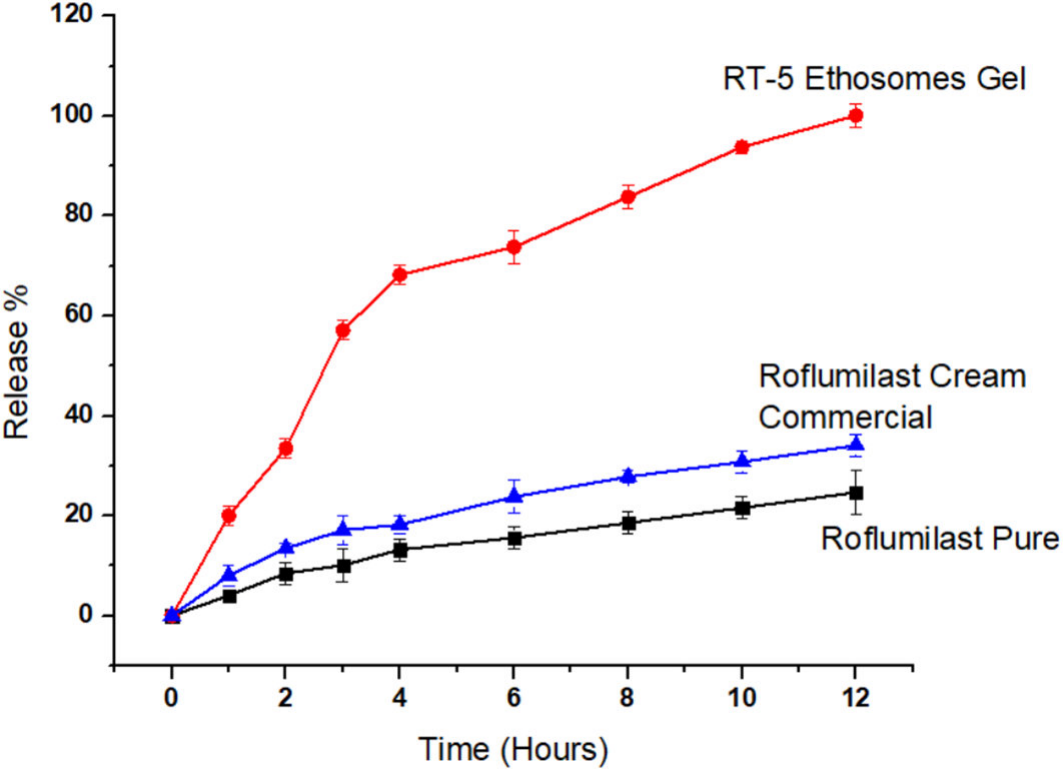
Drug release percentage of ethosomes (RT‐5) at pH 5.5.

The commercially available RT cream, on the other hand, had a significantly lower release profile, reaching about 34% by 12 h. Throughout the investigation, the pure RT exhibited the least amount of release, roughly 24% at 12 h. The poor water solubility of RT, which can limit dissolution and subsequent diffusion into the release medium, may be linked to the comparatively restricted release of pure RT.

The formulation’s capacity to enhance RT’s solubilization and dispersion within the phospholipid‐based vesicular system may be responsible for the increased release seen from the ethosomal gel. Greater drug availability in the release media may be facilitated by incorporating RT into nanoscale ethosomal vesicles, which offer a distinct microenvironment from that of the crystalline/free drug. Furthermore, the diffusion channel and release kinetics of RT can be altered by the phospholipid vesicular structure and gel matrix, leading to a more regulated and extended release profile. Rather than being a sustained‐release formulation, the commercially available cream was used as a marketing comparative. As a result, it offers a helpful comparison with the recently created ethosomal gel due to its comparatively lower and nonsustained release profile. In a similar vein, pure RT was used as an API control to determine the drug’s intrinsic release behavior. When compared to both pure RT and the commercial cream, the ethosomal gel’s release profile is noticeably different, suggesting that adding RT to the ethosomal gel system drastically alters its release properties. Overall, the results indicate that, in comparison to pure RT and the currently available cream, the proposed ethosomal gel can offer improved drug release together with an extended release pattern. The drug’s availability at the skin interface may also rise as a result of RT’s enhanced solubilization and dispersion throughout the ethosomal system. However, specific ex vivo or in vivo skin permeability tests would be necessary to confirm improved skin permeation or deposition.

### 4.10 Assay for Hemolysis

Since the ethosomes’ hemolysis assay can be performed quickly and reliably, it was used to determine if the material could be mixed with human blood [21, 30, 39, 47]. When blood comes into contact with a suspension of ethosomes, the hemolysis test measures the erythrolysis and hemoglobin dissociation that occur. For 12 h, the blood was supplemented with ethosome suspensions at concentrations of 50, 100, 200, and 500 mg/mL, as well as 0.05, 0.1, 0.2, and 0.5 mg/μL, respectively. The ethosomes suspension was measured in milligrams, and the blood sample was measured in milliliters, allowing calculation of the final concentration. The next step was to check the blood’s compatibility using a spectrophotometer. Triton‐X (100.00% lysis) served as the positive control, while PBS (not depicted) served as the negative control. Figure 9 shows that doses of 50, 100, 200, and 500 mg all yielded acceptable results in a 12 h blood compatibility test. The lysis proportion was less than 17% overall and less than 11% for both the 50 and 100 mg dosages. We conclude that ethosomes are safe to use and do not pose a threat to the blood. This could be because all of the chemicals were selected for their biocompatibility with blood, as per FDA rules, and RT and the other components were quite compatible with blood. Because of their malleable mechanical, physical, and biological characteristics, our research suggests that ethosomes may be employed for long‐term skin applications and have lasting hemostatic potential.

**FIGURE 9:**
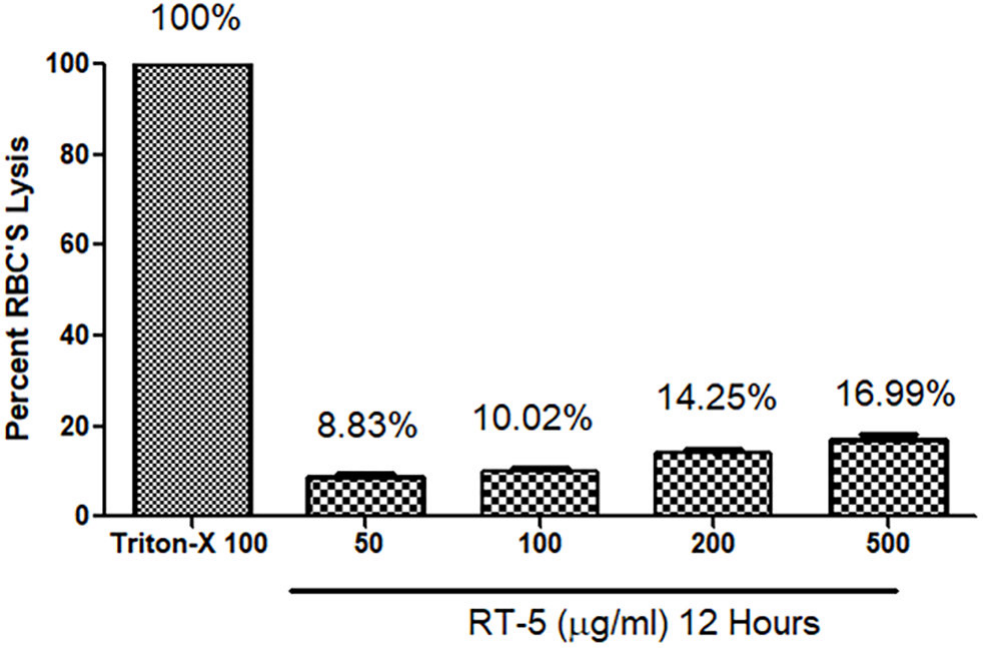
Hemolysis graph of ethosomes after 12 h.

### 4.11 In Vitro Permeability

An in vitro transdermal permeation study was performed to investigate and evaluate the transdermal permeation behavior of pure RT, commercial RT cream, and the optimized RT‐5 ethosomal gel using Strat‐M synthetic membrane under the above‐mentioned experimental circumstances. The receptor compartment was kept at 37 °C and swirled constantly at 50 rpm. 0.5 mL samples were taken from the receptor compartment at predetermined intervals of 1, 2, 3, 4, 6, 8, 10, and 12 h and replaced immediately with an equal volume of new receptor medium. The experiment was replicated 6 times. Figure 10 shows that the permeation characteristics of the three formulations of RT were significantly different across the 12‐h investigation. The highest cumulative penetration was shown by RT‐5 ethosomal gel, followed by commercially available RT cream, whereas the lowest permeability was displayed by pure RT throughout the research. The RT‐5 ethosomal gel showed the rapid initial penetration phase and about 25.5 ± 3% of RT permeated through the membrane in the first 1 h. The permeation rose gradually throughout the research and reached about 99.34% at 12 h. The commercial RT cream showed ~5.2 ± 2% penetration at 1 h and ~29% at 12 h. Figure 10 shows the penetration curve of the pure RT formulation was the lowest, with only about 12%–13% permeation at 12 h. The presence of pure RT and the commercial cream as comparator groups allow for an assessment of the role of the established ethosomal system in the observed improvement of penetration. The much decreased penetration of pure RT may be related to its low water solubility and subsequently limited dissolution and diffusion. Commercial cream penetration was higher than pure RT, suggesting that the standard formulation medium could augment drug availability to a certain degree. However, the much higher penetration seen with RT‐5 ethosomal gel suggests that the inclusion of RT into ethosomal system led to a significantly different permeation behavior compared to both the free drug and the marketed formulation. The improved penetration with RT‐5 may be ascribed to the nanosized vesicular architecture, better dispersion/solubilization of RT, phospholipid composition, and ethanol content of the ethosomal system. These formulation features may improve medication partitioning and diffusion through the Strat‐M membrane. The reduction in particle size achieved during formulation development may also result in greater medication availability and penetration. However, these mechanisms should be thought of as potential contributions and not as final explanations purely based on the present permeation experiment. The penetration profile of RT‐5 ethosomal gel was likewise in accordance with the improved release behavior shown in in vitro release research. The increased cumulative permeation with the ethosomal formulation as compared to pure RT as well as the commercial cream substantiates the ability of the proposed formulation to modify the delivery behavior of RT. However, as release and permeation investigations are separate experimental settings, the similarity of the two profiles should be regarded as supporting rather than indicative of a direct causative relationship. The results indicated that RT‐5 ethosomal gel showed much higher in vitro penetration across Strat‐M membrane than pure RT and commercially available RT cream under the test conditions. These data suggest that the ethosomal system can be a formulation strategy for enhancing the topical delivery of RT. Whether this increased membrane permeability translates to enhanced delivery through and/or retention in biological skin tissue will require further ex vivo skin permeation and skin‐deposition experiments.

**FIGURE 10:**
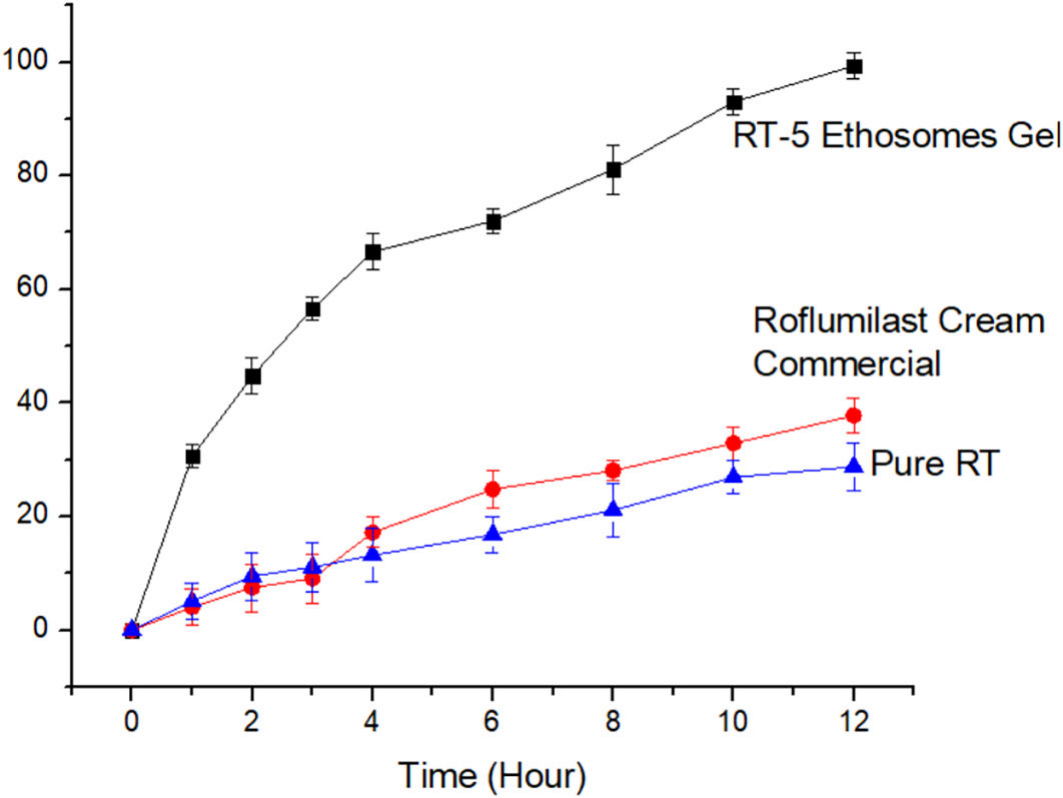
Permeation study using an artificial membrane at pH 5.5.

### 4.12 Rheological Properties of Ethosomes Gels

The concentration‐dependent constant‐shear‐flow curves of the ethosomes gels are depicted in Figure 11. The viscosities of ethosome gels of different concentrations displayed the properties of shear‐thinning pseudoplastic fluids when they were exposed to shear rates between zero and one thousand shear seconds per second. Various carobopol % values for the gel samples were tested for spreadability and were determined to be appropriate, as shown in Table 5. A strong correlation between concentration and viscosity was observed: the spreadability values of all ethosomes gel concentrations declined steadily, while the steady shear viscosity of all % gels increased from 1% to 1.5% and 2%. The rheological properties of the ethosomes gel, specifically its viscosity and spreadability, play a crucial role in both the ease of application and patient compliance. A gel with an appropriate viscosity ensures that the formulation is neither too runny nor too thick, making it easy to apply and distribute evenly on the skin. This optimal consistency improves patient satisfaction by facilitating smoother application and reducing the likelihood of irritation or discomfort.

**FIGURE 11:**
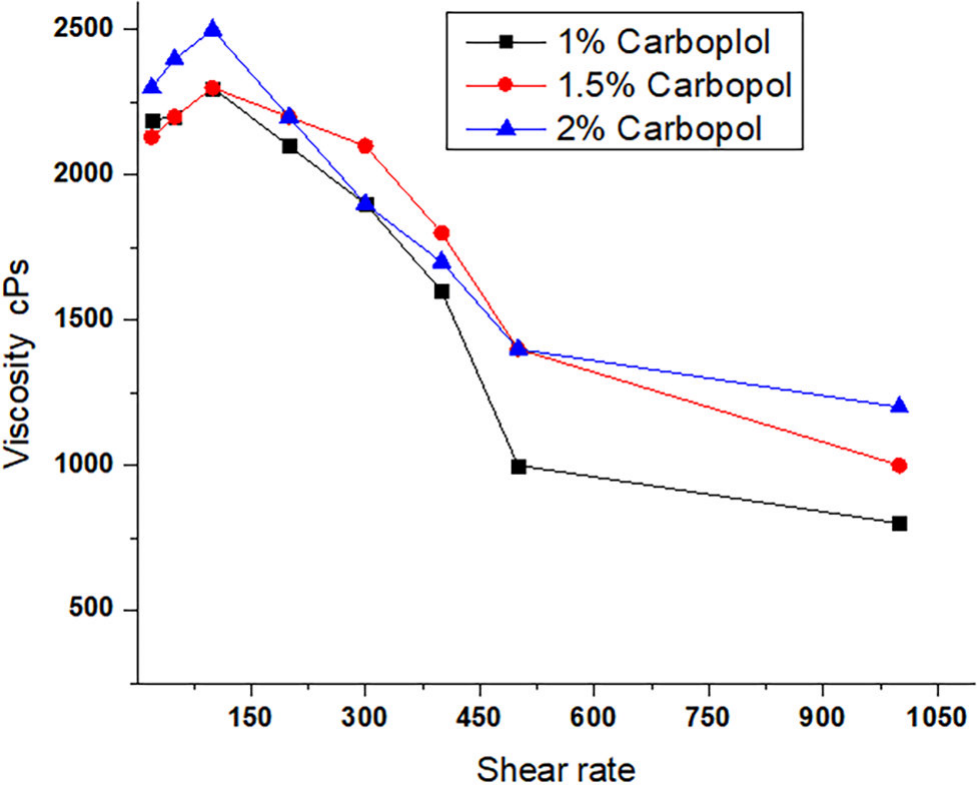
Shear flow curves of RT‐5 ethosomes gels with 1%, 1.5%, and 2% carbopol.

**TABLE 5:** Evaluation of ethosomes gel viscosity, spreadability, and pH.

Carbopol, % w/v	Viscosity using spindle S‐7	Spreadability, cm/5 min.	pH
1.0	74 ± 0.713	2.3 ± 0.12	5.3
1.5	88 ± 0.301	2.0 ± 0.19	5.7
2.0	99 ± 0.121	1.5 ± 0.11	5.6

### 4.13 The Spreading Ability and pH of Ethosomes Gel

The homogeneity and lack of air molecules in the gel were established by examination. It was found that all of them had pH levels ranging from 5.3 to 5.7. Gels produced from engineered ethosomes spread well with minimal shearing force, as measured in tests of spreadability. A spreadability range of 2.3 ± 0.12–1.5 ± 0.11 cm/5 min was demonstrated for an ethosomes gel containing RT. In addition, we determined that the ethosomes gel had a viscosity ranging from 26,200 ± 623 cPs to 29,100 ± 184 cPs. The results showed that the viscosity was directly related to the percentage of polymeric material.

#### 4.13.1 Physical, Chemical stability and microbial assessment

After transforming an RT‐5 solution into a gel formulation, we measured its stability and total aerobic microbial count (TAMC). The skin was the intended target of the formulation’s development. The gel underwent stability testing by being kept at 40 °C for 6 months in a stability chamber. The gel, order, appearance, pH, and viscosity were tested on the first day and at 3, and 6 months thereafter. The gel’s pH ranged from 6.3 to 6.5, which is considered suitable for skin care treatments. At 25 °C, the formulation’s viscosity was 79–80 cpi, which is suitable for cutaneous application as well. Before the produced gel is converted into a patient‐sized formulation, it must meet all USP 43 standards [48]. Additional checks were made on the gel’s microbiological characteristics. For up to 6 months, TAMC, TYMC, Staphylococcus, and were studied in gel. Following the method outlined by Ballal, N. V., et al. [42], the plates were prepared using Sabouraud dextrose agar and nutritional agar. The plates were incubated in Memmert incubators for 72 h at TAMC and for 7 days at different temperatures (32 °C  ± 2  and 22 °C  ± 2). The results shown in Table 6 are quite satisfactory until the 6th month.

**TABLE 6:** Stability results after 6 months at 40 °C.

Test	Results			Limits
	Initial	3rd Month	6th Month	
Order	Complies	Complies	Complies	Characteristic order
Appearance	Complies	Complies	Complies	Clear
pH	5.5	5.3	5.4	5–6
Viscosity	91cpi	90cpi	90cpi	75–105cpi
Total aerobic microbial count (TAMC)	<5 cfu/g	<5 cfu/g	<5 cfu/g	200 cfu/g
Total combined yeasts and molds count (TYMC)	<5 cfu/g	<5 cfu/g	<5 cfu/g	20 cfu/g
Tests for absence of specified microorganisms				
*Staphylococcus aureus*	Absent/g	Absent/g	Absent/g	Should be absent/mL
*Pseudomonas aeruginosa*	Absent/g	Absent/g	Absent/g	Should be absent/mL

The assay results decreased gradually with the increase of storage time, and the fitted regression equation was Assay = 101 − 0.153 × Month. The predicted shelf life of the formulation was approximately 24.37 months based on the lowest specification limit of 95%. The results show satisfactory retention of medication content over the studied period. Besides medication content, the physical stability of the formulation during long‐term storage would be further informed by the physical integrity of the ethosomal vesicles, including any changes in vesicle features.

For regulatory approval, this study’s findings regarding the formulation’s stability, effectiveness, and safety are vital. Contributing to the regulatory framework for innovative medication delivery systems, these findings align with the expectations of regulatory authorities such as the FDA and EMA. To speed up the approval process, the detailed information provided could be crucial (Figures 12 and 13).

**FIGURE 12:**
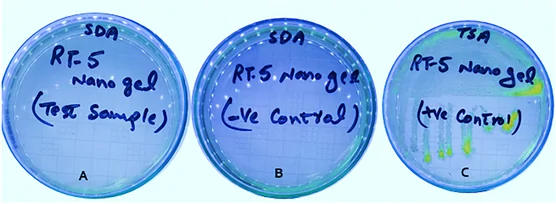
Microbial burden RT‐5 gel after 6 months Plate A is test sample RT‐5, plate B is negative control and plate C is positive control.

**FIGURE 13:**
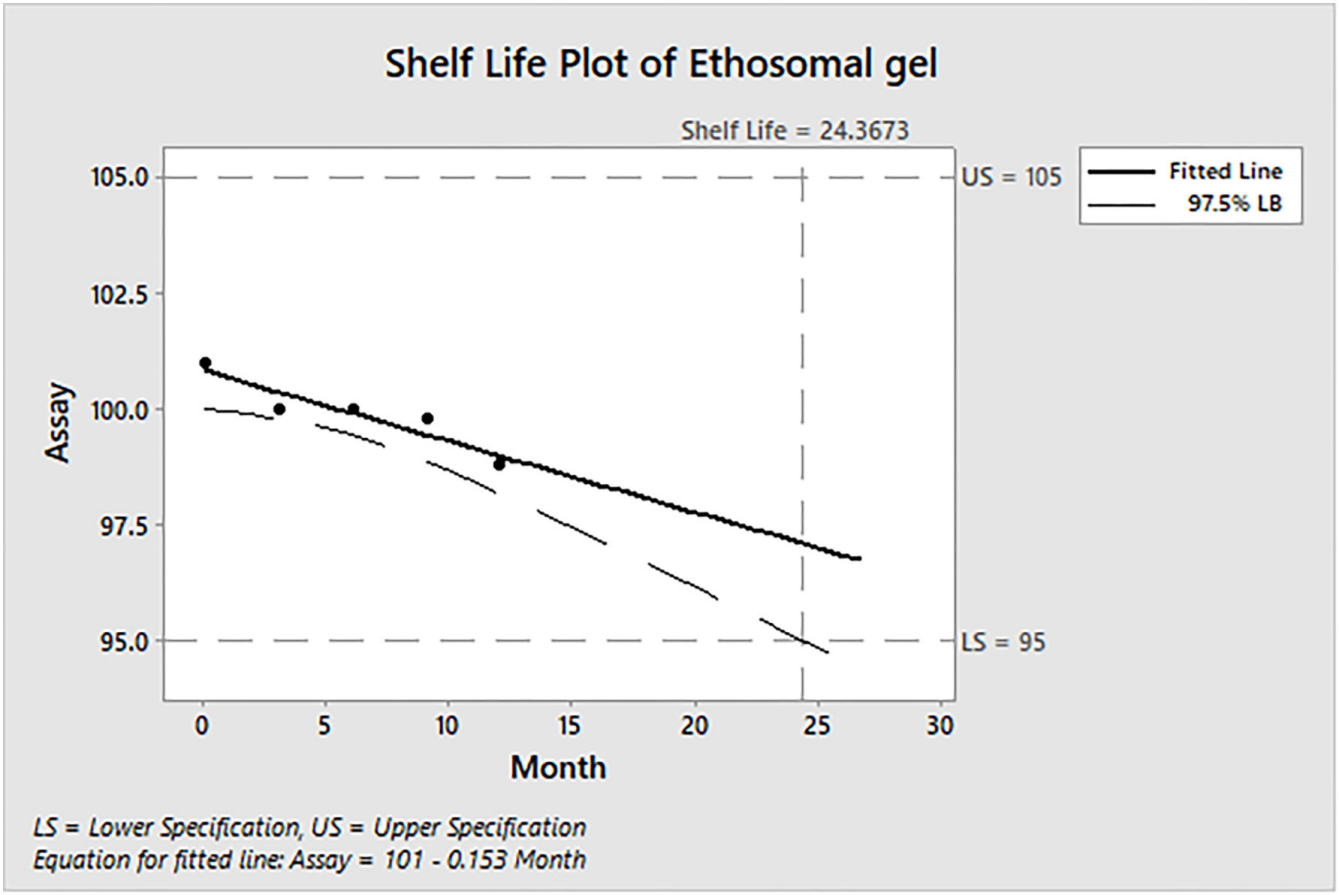
Stability trend for 24 months.

## 5 Conclusion

Our results show that adding RT to ethosomes greatly improves their transdermal penetration, stability, and solubility. Electrostatic interactions, hydrogen bonds, and the encapsulation of molecules inside the ethosomes’ vesicles are the main causes of this enhanced entrapment. Since RT is encapsulated, there is less direct exposure to the aqueous environment, reducing the risk of degradation and increasing stability. The developed formulation represents a promising topical delivery system, while its therapeutic efficacy and anti‐inflammatory mechanism require further investigation through appropriate in vitro cell line and subsequent ex vivo/in vivo studies. The ethosomes formulation is a safer, easier substitute for traditional topical systems as it is completely water‐based and doesn’t require any additional excipients. The formulation is now a ready‐to‐use topical gel, having been successfully scaled up and refined. The ethosomes gel showed up to 3 times the medication release and skin penetration of commercially available 0.3% RT lotions.

## Author Contributions

Conceptualization, original draft writing, reviewing, and editing: Yasir Mehmood, Syeda Momena Rizvi, Atika Javed, and Rida Siddique. Formal analysis, investigations, funding acquisition, reviewing, and editing: Hamza Rafeeq and Hira Shahid. Resources, data validation, data curation, and supervision: Ousman B. Mahamat, Hafiz Muhammad Zohaib, Khalid S. Almaary, Gamal A. Shazly, Musa A. Said, and Turki M. Dawoud.

## Funding

This work is financially supported by the Ongoing Research Funding Program, King Saud University Riyadh, Saudi Arabia (ORF‐2026‐189).

## Ethics Statement

The protocols used in the current study were approved by the Ethical Review Board of Rashid Latif College of Pharmacy (IRB No. RLCP/EP/12/2024, in accordance with the World Medical Association’s Declaration of Helsinki.

## Consent

Informed consent was obtained from the participants.

## Conflicts of Interest

The authors declare no conflicts of interest, financial, or otherwise.

## Data Availability

All data generated or analyzed during this study are included in this published article.
